# Dr. Kinglake on Obstetric Practice, in Reply to Dr. Merriman

**Published:** 1816-05

**Authors:** 


*For the London Medical and Physical Journal.*

### Dr. Kinglake *on Obstetric Practice, in Reply to Dr. Merriman.*

YOUR correspondent, Dr. Merriman, has reserved his comments on my observations on obstetric practice until he imagined that he could wield them with *pedagogal* severity. He has kept aloof until he fancied he could combat my opinion with all the crushing authority of an *introductory lecture* on the indispensable necessity of man-midwifery practice. Many of his pupils will probably recognize, if not word for word, quotation for quotation, at least the general form and import of that gentleman's solemn commendation of his favourate occupation, in the philippic with which he has thought proper to asperse my observations on a subject of momentous importance to humanity.

It is Dr. Merriman's province, as it appears to be his darling fort, to espouse whatever habit and adoption have authorised in the practice of man-midwifery. He has cited, adduced, and calculated on points that have never been disputed by me. That placental and various other presentations actually occur, and that these cases require scientific aid, no one has ever doubted. My objection is founded on such presentations being unnatural, and that things so circumstanced are not likely to be often happening. My principle rests on the general sufficiency of nature, in utter defiance of all the speculations, and what appear to me to be the gratuitous conclusions of art. Dr. Merriman, reversing the *literal* import of his *name*, affects to be *gloomy*, even to abhorrence, at some of my expressions. He says, they are not "gentlemanly," and that they even "charge wilful murder!" Now this imputation is neither *merry*, nor light, nor defensible. It evinces an irrascibility, a "splenetic effusion," to adopt the doctor's own phrase, at any thing savouring of intrusion into a province of medical reasoning which he assumes to be exclusively his own, and that of those who are associated in his views of establishing the practice of man-midwifery on the legitimate authority of correct principles. I am, then, at issue with Dr. Merriman on the correctness of his obstetric principles, on the practice he founds on them, on the authorities they afford, and on the effects of their confident adoption.

To infer a "charge of murder" on the conduct of respectable, nay, highly skilful persons, according to the precepts of professed teachers, would be to criminate the *learner* for the errors of the *teacher;* it would be to proscribe the application to practice of what is affirmed to be indispensably requisite. Were I disposed to impute the charge referred to, it should not be against instructed man-midwives, but against the asserters and defenders of the established system. But this would be wrong: it would be travelling far beyond the bounds of candour, of liberality, and of justice. No such charge could justly lie against either Dr. Merriman or the host of authors and preceptors whom he has triumphantly cited. They have unquestionably the most honourable and humane intentions in all they maintain on the subject. The fatal error common to science, and to every species of adopted opinion, is a rooted inveteracy against all innovation; and when the attempt to amend is directed against a specific number of advocates, a sort of *esprit de corps* is manifested, and an impatience, nay, a virulence, discovered at at being contradicted or questioned, that has more the aspect of "craft" (I pardon the repetition of the epithet) than of undisguised frankness, and an unfeigned disposition to reconsider the disputable point. It is at present the authorised practice in man-midwifery to employ the various resources of the obstetric art in circumstances that are at least equivocal for a reference to that aid; or rather, the latitude that necessarily must be conceded to the intelligent interpreter of these general obstetric warranties is, in my apprehension, inseparably accompanied with frequent occasions for distrust and regret for the inevitable consequences.

Dr. Merriman should be seriously *grave* when he solemnly charges another who has feelings as well as himself with an idea of suspecting any human creature of deliberate murder! Is it "gentlemanly," is it decorous, nay, is it not truly "disgusting," for a person of Dr. Merriman's obstetric reputation to be borne away with a violence that would be inexcusable in one of his rawest tyros, because points connected with his professed practice are questioned and impugned? If a *hue and cry* about "murder" and other intemperate nonsense are to restrain the just liberty of remarking on principles and practice in any department of the medical profession, there would be an end put to all independant and conscientious investigation;—the tyranny of opinion and adoption would usurp the place of discussion and correction; and erroneous prejudices would have a chance of being sanctioned, if not by "craft," at least by clamour and groundless opposition.

Before Dr. Merriman again assumes an ungoverned rage in defence of what I have no doubt he sincerely believes to be correct, he should remember the fallible nature of all human reasoning, he should recollect that scarcely a hundred years have yet elapsed since the *inflammatory* character of the small-pox was denied and reprobated, and that the heating treatment of that disease has had its hecatombs of victims to its delusive correctness. Had Dr. Merriman flourished in the beginning of the eighteenth century, and been as much connected with *variolous* as he now is with *obstetric* practice, his persuasion of the correctness of the treatment then adopted for that disease would have been as firm and unshaken, and his expressions as confident and ungracious, as they are in support of his wedded attachment to modern accoucheurship. Does Dr. Merriman, in the visionary accuracy of all his obstetric views, and in his unsparing imputations on any one who would question them, believe that any person condemning the heating treatment of smallsmall-pox, and its destructive tendency, would be charging "wilful murder" on the unoffending zealots of that treatment. It was the delusion of principle that misled to the hurtful practice; and it is against what appears to me to be in some respects a similar delusion that I have directed the objections which Dr. Merriman has so contumaciously resisted.

The yellow-fever, as it was called, that thinned the population of Philadelphia, and its environs, towards the close of the last century, was held to be of a typhoid character, and was treated with tonics and stimulants. This treatment was authorised by the established principles of the day applicable to that description of disease: the results of the practice, however, proved much more baneful than the spontaneous course of the distemper. Dr. Rush, led back by accurate observations from the seductive influence of system and prejudice, reversed the treatment, and, in so doing, annulled the destructive power and horror of the complaint. Bleeding and purgatives, to an un-heard-of extent, superseded the use of bark, wine, and the whole tribe of anti-putrescent stimulants. Thousands are yet alive to attest the happy, the intelligent controul under which that exterminating disease was brought by the contrasted treatment that was instituted. If the zeal with which Dr. Merriman advocates whatever he has proposed and adopted in the obstetric art had maintained its ascendancy in the stimulating mode of treating the American disease under consideration, what would have ensued? Thousands now existing would have been added to the defunct list; but it may be justly presumed that the enlightened, the judicious, nay, the skilful practitioners who had so treated them, would neither have been charged, nor could have been morally held as guilty of "murder." The advocates of the stimulating treatment were told by a contrary experience, as well as by the honest injunctions of the humane, that they were wrong, that the treatment they pursued would be mortally injurious; but they were unconvinced of their error, and persisted in it, under a conscientious persuasion that they were correct; precisely in the same manner as modern accoucheurs persist in the practice of interfering, in anticipating, and occasionally in perverting, the regular course of Nature, not to "murder," but to assist *secundum artem*, and according to the authorities of doctrines confidently taught and implicitly adopted.

The cooling treatment of gout, to which I some years since drew the public attention, has been also reprobated as fraught with with the most deadly evils. Time has greatly diminished the early asperity with which it was opposed. Some scurrilous reviewers, indeed, on that occasion, went the length of Dr. Merriman in charging the crime of "murder" on any one who should have the unfeeling audacity and hardihood to authorise and defend such an altered treatment of the disease. Had I been terrified by inconsiderate alarmists, or rebuffed by "ungentlemanly" coarseness, and criminal accusation, the salutary effects of the practice would not have been known as they now are, and the pride and folly of ancient errors and prejudices on the subject would have triumphed over a benevolent endeavour to introduce an improved mode of practice in that painful and undermining disease. The perfectionist in morals, as well as in medical physics, may muse on the ideal completeness, the finished accuracy, of his views; but the liberal and unprejudiced observer will discover defects at almost every point, which he will feel bound to expose and comment on without fear or apprehension from those who indignantly resist every attempt to storm the fortress of established and venerated abuses.

If Dr. Merriman and his obstetric fraternity regard themselves as privileged in proposing and in insisting on certain auxiliary schemes in aid of Nature's parturine insufficiency, and if they protest against all comments on those assumptions, as if they were consecrated by the natural order of things, there is an end of all freedom of discussion; adverse arguments must succumb under *corporate* law, and he who dissents must compound by being branded with imputations of "inexperience," "ignorance," "disingenuousness," "ungentlemanly" expressions, &c. for disturbing such settled and indisputable authority.

Dr. Merriman may take his stand with art, I will patiently take up my abode with nature; and, though the infirmities of the latter may be at times usefully assisted by the former, it will not be in the power of any one to convince me that the generative system of animal life is so constituted as to require the almost incessant watchings and assistance of adventitious power to further and extend the advantages of parturition.

*Taunton;*
*April* 10, 1816.

